# Shared environment and colorectal cancer: A Nordic pedigree registry‐based cohort study

**DOI:** 10.1002/ijc.34148

**Published:** 2022-06-22

**Authors:** Rahma Elmahdi, Elna C. M. Wennerström, Mikael Andersson, Jan Wohlfahrt, Mads Melbye, Eero Pukkala, Maria Hortlund, Kaisa Silander, Kyösti Sutinen, Tine Jess, Joakim Dillner

**Affiliations:** ^1^ Department of Clinical Medicine, Center for Molecular Prediction of Inflammatory Bowel Disease (PREDICT) Aalborg University Copenhagen Denmark; ^2^ Department of Epidemiology Research Statens Serum Institut Copenhagen Denmark; ^3^ Department of Clinical Medicine University of Copenhagen Copenhagen Denmark; ^4^ Center for Fertility and Health Norwegian Institute of Public Health Oslo Norway; ^5^ Faculty of Medicine, Department of Public Health, K.G. Jebsen Center for Genetic Epidemiology Norwegian University of Science and Technology Trondheim Norway; ^6^ Faculty of Social Sciences Tampere University Tampere Finland; ^7^ Finnish Cancer Registry Institute for Statistical and Epidemiological Cancer Research Helsinki Finland; ^8^ LINK Medical Research Malmö Sweden; ^9^ Finnish Institute for Health and Welfare Helsinki Finland; ^10^ Department of Gastroenterology and Hepatology Aalborg University Hospital Aalborg Denmark; ^11^ Medical Diagnostics Karolinska Karolinska University Hospital Stockholm Sweden; ^12^ Department of Laboratory Medicine Karolinska Institutet Stockholm Sweden

**Keywords:** cohort study, colorectal cancer, pedigree registries

## Abstract

Risk of colorectal cancer (CRC) increases in relatives of patients with CRC. The extent to which this is attributable to genetic predisposition or shared environment is unclear. We explored this question using nationwide cohorts from Denmark, Finland and Sweden. From 1977 to 2013, we identified 359 879 individuals with a CRC diagnosis and 2 258 870 of their relatives who we followed for CRC incidence. We calculated standardized incidence ratios (SIR) and 95% confidence intervals (CI) for CRC in individuals with an affected relative. We used nationwide household and pedigree data along with national SIR estimates to calculate risk ratios (RR) for the contribution of shared household environment, childhood environment and genetic relationship to CRC risk in those with an affected relative. SIR of CRC was increased for individuals with an affected relative, across all countries and ages. For those with an affected parent, the SIR was 1.65 (95% CI: 1.61‐1.69), 1.98 (95% CI: 1.87‐2.09), for those with an affected sibling and 2.14 (95% CI: 1.84‐2.49) for those with an affected halfsibling. In those <65 years old, shared childhood (RR: 1.41, 95% CI: 1.26‐1.57) and household (RR: 2.08, 95% CI: 1.25‐3.46) environments were significantly greater contributors to familial risk of CRC than genetics (RR: 0.88, 95% CI: 0.53‐1.46). This large‐scale Nordic population‐based study of excess risk of CRC among relatives of those with CRC addresses the difficult disentangling of shared environment from genetic predisposition in the heritability of CRC. We found shared environment to be the most important contributor to CRC risk.

## What's new?

The risk of colorectal cancer increases in relatives of colorectal cancer patients. Because several known environmental risk factors for colorectal cancer are commonly clustered within families, such as obesity, smoking and other diet or lifestyle factors, the extent to which the excess risk is due to genetic predisposition or a shared familial environment remains unclear. In this joint Nordic cohort study using nationwide population‐based cancer and pedigree data of over 2.25 million individuals, the authors found that a familial shared environment was a greater contributor to the excess risk of colorectal cancer among first‐degree relatives than shared genetics.

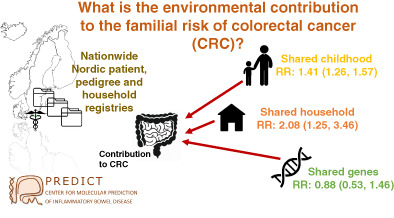

## 1 BACKGROUND

Colorectal cancer (CRC) is the third most common cause of cancer worldwide with a projected increase of more than 60% to 2.2 million new cases by 2030.^1^ In the Nordic countries, CRC is also the third most common cause of cancer‐related mortality.^2^

Approximately 5% of all CRCs occur in persons with one of two monogenic hereditary syndromes (Familial Adenomatous Polyposis and Lynch syndrome/hereditary nonpolyposis colorectal cancer).^3^ In addition, recessive mutations are further implicated in the progression of normal colonic epithelium to invasive carcinoma, which make up the >90% of incident cases.^4^

Recent studies report a greater than 2‐fold increased risk of CRC in individuals who have one or more relative with CRC.^5^, ^6^ This increased risk also appears to be maintained in families without an identified monogenetic cause, and in some Nordic twin‐studies, this has been found to be even higher.^7^, ^8^ It remains unclear what the contribution of shared environment is to this observed increased risk of CRC in first‐degree relatives, as several known environmental risk factors for CRC are also commonly clustered within families, for example, obesity, smoking and other diet and lifestyle factors.

Nordic countries have an infrastructure enabling large‐scale and reliable epidemiological research, due to their population‐based and comprehensive health data registry systems, which are linkable using unique personal identification codes. In the present joint Nordic collaborative research study, we constructed a large international cohort composed of all patients diagnosed with CRC and their relatives in Denmark, Finland and Sweden who could be followed for several decades. Our aim was to explore the risk of CRC in the family members of those with a CRC diagnosis, examining the extent to which shared childhood and household environment contributed to this risk.

## 2 METHODS

### 2.1 Identification of CRC

Data for CRC diagnosis are available for our purposes for Denmark, Finland and Sweden from 1977,^9^ 1953^10^ and 1958,^11^ respectively. Over the follow‐up period, CRC diagnosis is identified using the International Classification of Diseases seventh revision (ICD‐7), and tenth revision (ICD‐10), and the third edition of the International Classification of Diseases for Oncology (ICD‐O‐3) in each of these registries. For Denmark, CRC diagnoses are extracted from the Danish Cancer Registry (DCR)^12^ where CRC was coded using ICD‐10 with code C18 for colon cancer, and C19‐C20 for rectal cancer from 1978. In Finland, information on CRC diagnosis was collected from the Finnish Cancer Registry (FCR), which uses ICD‐O‐3.^13^ Here, colon cancer is coded as C18 and rectal cancer as C19‐C20. In Sweden, information on CRC diagnoses was collected from the Swedish Cancer Registry (SCR) using ICD‐7 codes (colon cancer: 153; and rectal cancer: 154).^14^ Due to different practices in CRC diagnosis coding over time (specifically historic use of recto‐sigmoid diagnostic code in Denmark from 1977 to 1978), it was not possible to disaggregate colon cancer diagnoses from rectal cancer diagnoses across all countries.

### 2.2 Data on relatives

**FIGURE 1:**
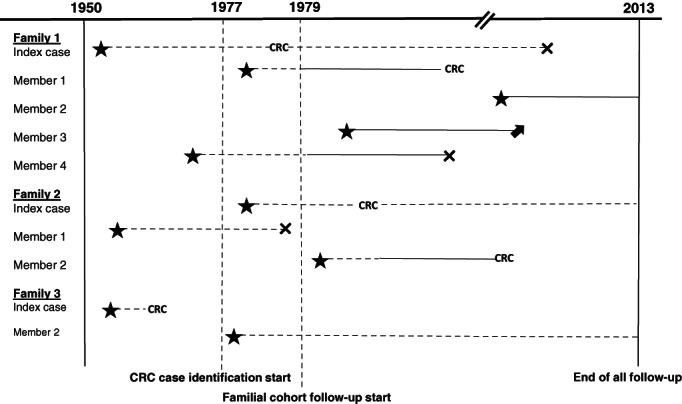
The built cohort: Index cases and family cohort with case identification and contribution to follow‐up time. Key: 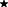, Birth; 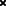, death; 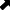, emigration; 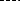, no contribution to follow‐up; 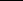, contribution to family cohort follow‐up; CRC, colorectal cancer diagnosis

Using the national pedigree registries of Denmark, Finland and Sweden, relatives were classified as first‐degree relatives (parent, child and sibling) or as second‐degree relatives (halfsibling, grandparent, grandchild, aunt/uncle and nephew/niece). Relatives without a genetic link to cases (eg, adopted children where there is no first or second degree relation) were excluded from the family cohort. The year of pedigree registry start up and national coverage of pedigree registries differed between countries; 1977 in Denmark, 1954 in Finland and 1970 in Sweden. National cancer incidence reports from Sweden and Finland were aggregated over 5‐year periods until 1979. Inclusion into the study for relatives of index patients (patients with a CRC diagnosis) therefore began in 1979, the point where comparable data on annual cancer incidence and pedigree registries were available in all three countries (Figure 1).

In Denmark, information on parents of individuals born 1968 and onward, was collected from the Danish Civil Registration System (DCRS) to identify relatives.^9^ The parental link allowed for identification of both first‐ and second‐degree relatives, alive and living in Denmark from 1968 onwards. These individuals were linked to the DCR, via a unique Personal Identification Number.

Data on familial relationships of individuals born 1953 and onward for Finland were collected from the Finnish Civil Registration System (FCRS). The parental link was used for identification of first‐, and second‐degree relatives. The records of parental links as registered in the FCRS were first created for all parent‐child pairs that shared an address in 1973, and after that links to parents were created for all residents in Finland born after 1973.^15^ Individuals in all registries are linkable via the unique Personal Identity Code.

In Sweden, all first‐ and second‐degree relatives are identifiable through the Swedish Multi‐generation Registry.^16^ In some cases, the type of sibling (full or half) is not reported. These individuals were only included in the overall “Any Relative” analysis and excluded elsewhere.

### 2.3 Design and follow‐up

First, a CRC index cohort of individuals diagnosed with CRC was created and, subsequent to this a family cohort was overlaid, indicating the relationship to the index case. The CRC index cohort included all individuals with a CRC diagnosis, and the family cohort consisted of individuals having a first‐ or second‐degree relative with a previous CRC diagnosis. Identification of index cases commenced 1 January 1977 for incident cases of CRC. In the family cohort, follow‐up commenced from 1979 (the point at which family linkage becomes available in all included countries and all CRC incidence data is disaggregated by year). Individuals born after the index CRC diagnosis were followed from date of birth to point of CRC diagnosis, emigration, death, end of study (31 December 2013 for Denmark and Finland, and 31 December 2012 for Sweden), or whichever came first (Figure 1).

### 2.4 Statistical analysis

Standardized incidence ratios (SIR) with 95% confidence intervals (95% CI) were calculated for the risk of CRC among first‐ and second ‐degree relatives of the CRC index patients by comparing the number of observed CRC cases to the expected number of CRC cases nationally. The expected number of CRC cases were calculated as the number of person‐years of the relatives multiplied by national CRC rates in the same sex, age and time‐period for the corresponding country.

### 2.5 Shared familial environment and genetics model

To further explore the degree to which familial aggregation of CRC was due to genetic or environmental factors, a simplified shared environment and genetic model was applied. The relative risk (RR) of CRC, in individuals with a family history compared with those without a family history, was assumed to be the product of three components; a genetic component (G), a shared household environment component (a shared household with any affected relative; H) and a shared childhood environment component (a shared household with an affected sibling or halfsibling; C). Shared household and shared childhood environments are therefore identified through a combination of the civil registries and age of related individuals. The genetic component includes first‐degree relatives' genetic component (G1) and second‐degree relatives' genetic component (G2). The relationship between G1 and G2 has previously,^17^ been described as G2 = √G1. We therefore defined parent, child and sibling relations as “first‐degree relatives” and halfsibling relations as “second‐degree relatives.” In order to account for the effect of time period on environment, only half‐siblings were used to calculate the genetic component of second‐degree relatives for the purposes of this analysis. Thus, the RR for having an affected parent or child is modeled as G1*H, the RR for having an affected sibling is modeled as G1*H*C, and the RR for having an affected halfsibling is modeled as G2*H*C. G1, H and C are derived from the estimated SIR, according to country, age and family relation by inverse‐variance‐weighting using the PROC NLMIXED procedure in SAS. The shared childhood environment component is a model‐based estimate of how much higher an individual's risk is when having an affected relative with shared childhood environment, but without shared household environment or genetics, compared with the expected risk in the population. The same type of interpretation can be applied to the shared household environment and the genetic component. Important assumptions underlying the model are that the assumed relationship between G1 and G2, and the multiplicative structure are good approximations of environmental and genetic contributions, and finally that interactions between the effects of genetic, shared childhood environment and shared household environment can be ignored (i.e. exclusion of potential epigenetic effects).

A sensitivity analysis was undertaken using the same model as above to assess the risk of CRC in those with a shared household compared with a shared childhood environment, independent of the shared genetic component. This was undertaken to assess the degree of effect‐modification by age, sex and cancer site (where this data was available) excluding the contribution of the shared genetic component, with risk ratios and 95% CI calculated for each. All statistical analysis was performed using SAS (version 9.4, SAS Inst Inc., Cary, NC).

## 3 RESULTS

Baseline characteristics of the study cohort by participating country are displayed in Table 1. A total of 359 878 individuals (121 319 from Denmark, 71 276 from Finland and 167 283 from Sweden) had at least one diagnosis of CRC identified in the cancer registries and these formed the index cohort. Among index cases, 97 326 had no identifiable relative in the pedigree registries during the study follow‐up period. The remaining cases were linked to 2 258 870 relatives (384 993 from Denmark, 393 986 from Finland and 1 479 891 from Sweden), which made up the family cohort. Of these, 262 488 were first‐degree relatives (71 901 from Denmark, 54 363 from Finland and 136 224 from Sweden) and 235 521 were second‐degree relatives (63 643 in Denmark, 47 255 in Finland and 124 623 in Sweden). There was a total 1.77 million, 2.07 million and 5.99 million person year contribution from Denmark, Finland and Sweden, respectively. Complete sample sizes and the crude incidence rates of colorectal cancer per 100 000 person‐years is presented by nature of relation (e.g., parent, child, sibling and halfsibling), age at start of follow‐up, sex and country in Table S1.

**TABLE 1:** Baseline characteristics of participants by country

	Country		
	Denmark	Finland	Sweden
Index cohort			
All			
N	121 319^a^	71 276	167 283
Birth cohort, min‐max years	1875‐2001	1880‐2004	1900‐2005
Birth cohort, median year (q1‐q3)	1925 (1914‐1936)	1927 (1916‐1939)	1924 (1914‐1935)
Person years	618 537	418 940	1 049 511
CRC cases (new CRC during follow‐up)			
N (%)	2397 (2.0)	694 (1.0)	4406 (2.6)
Birth cohort, min‐max years	1888‐1979	1887‐1984	1900‐1989
Birth cohort, median year (q1‐q3)	1927 (1918‐1937)	1925 (1917‐1935)	1923 (1915‐1932)
Days before subsequent CRC diagnosis, median (q1‐q3)	1979 (882‐4322)	2984 (1127‐6179)	1826 (546‐4567)
Diagnosis codes			
Colorectal cancer (CRC)	ICD10: C18‐C20	ICDO3: C18‐C20	ICD7: 1530‐1539, 154, 1540, 1541, 1548
Colon cancer	ICD10: C18	ICDO3: C18	ICD7: 1530‐1539
Rectal cancer	ICD10: C19, C20	ICDO3: C19, C20	ICD7: 154, 1540, 1541, 1548
Unspecified	‐	‐	‐
Family cohort^b^			
All			
N	384 993^a^	393 986^c^	1 479 891
Birth cohort, min‐max years	1880‐2013	1880‐2013	1900‐2012
Birth cohort, median year (q1‐q3)	1984 (1965‐1995)	1978 (1961‐1990)	1981 (1963‐1997)
Person years	4 407 439	5 156 587	20 949 513
CRC cases			
N (%)	1266 (0.3)^a^	1572 (0.4)^c^	6649 (0.4)
Birth cohort, min‐max years	1895‐2000	1897‐2002	1900‐2001
Birth cohort, median year (q1‐q3)	1951 (1938‐1958)	1948 (1940‐1955)	1942 (1936‐1949)
Index cohort			
All			
N	121 319^a^	71 276	167 283
Birth cohort, min‐max years	1875–2001	1880‐2004	1900‐2005
Birth cohort, median year (q1‐q3)	1925 (1914–1936)	1927 (1916‐1939)	1924 (1914‐1935)
Person years	618 537	418 940	1 049 511
CRC cases (new CRC during follow‐up)			
N (%)	2397 (2.0)	694 (1.0)	4406 (2.6)
Birth cohort, min‐max years	1888–1979	1887–1984	1900‐1989
Birth cohort, median year (q1‐q3)	1927 (1918–1937)	1925 (1917–1935)	1923 (1915‐1932)
Days before subsequent CRC diagnosis, median (q1‐q3)	1979 (882–4322)	2984 (1127–6179)	1826 (546‐4567)
Relatives of index cohort			
No relatives, N (%)	49 388 (40.7)	16 913 (23.7)	31 025 (18.5)
At least 1 first degree, N (%)	71 901 (59.3)	54 363 (76.3)	136 224 (81.4)
At least 1 second degree, N (%)	63 643 (52.5)	47 255 (66.3)	124 623 (74.5)
At least 1 third degree, N (%)	1167 (1.0)	2299 (3.2)	79 204 (47.3)
Median number of relatives (q1‐q3)	2 (0–2)	4 (1–9)	8 (2‐14)

Abbreviations: CRC, colorectal cancer; N, number; q, quartile range.

^a^ In Denmark relatives need to be alive after 1977 to be included.

^b^ Counting cancer cases as number of diagnoses.

^c^ CRC diagnosis of relatives may occur before start or after end of index‐persons follow‐up (in instances where a person may be a family member for an index case but also an index case for a new family cohort).

The SIR of CRC in those with any relative with CRC was 1.57 (95% CI: 1.54‐1.60) compared with the respective reference population (Table 2), regardless of type of family relation (parent, sibling and halfsibling) across all three countries, and this was especially pronounced in those below the age of 65 (SIR: 1.70, 95% CI: 1.66‐1.75). National differences in the SIR for CRC were observed for individuals below 65 years with an affected relative, with a statistically significantly increased SIR in Finland (SIR: 1.99, 95% CI: 1.88‐2.11), compared with that for Denmark (SIR: 1.78, 95% CI: 1.67‐1.90) or Sweden (SIR: 1.62, 95% CI: 1.57‐1.67; *P* < .0001). This increased risk also appeared in those aged 65 or older, with the SIR of CRC in any relative of an affected individual being 1.53 (95% CI: 1.40‐1.68) in Finland, 1.49 (95% CI: 1.35‐1.64) in Denmark and 1.38 (95% CI: 1.33‐1.43) in Sweden, however, the difference in increased SIR between countries was not significant for this age group (*P* = .06).

**TABLE 2:** Standardized incidence ratio (95% CI) for colorectal cancer in Denmark, Finland and Sweden by Relative with CRC and age (below 65 years and 65 years and over)

Relative with CRC	All countries SIR (95% CI)	Denmark SIR (95% CI)	Finland SIR (95% CI)	Sweden SIR (95% CI)	*P*_diff_ by country
All					
Any relative	1.57 (1.54‐1.60)	1.68 (1.59‐1.77)	1.83 (1.74‐1.92)	1.50 (1.47‐1.54)	<.0001
Parent	1.65 (1.61‐1.69)	1.67 (1.57‐1.78)	1.97 (1.86‐2.09)	1.58 (1.54‐1.63)	<.0001
Sibling	1.98 (1.87‐2.09)	2.55 (2.06‐3.15)	2.38 (2.07‐2.73)	1.88 (1.77‐2.00)	<.001
Halfsibling	2.14 (1.84‐2.49)	2.15 (1.50‐3.10)	2.35 (1.84‐3.00)	1.99 (1.58‐2.49)	.61
Below 65					
Any relative	1.70 (1.66‐1.75)	1.78 (1.67‐1.90)	1.99 (1.88‐2.11)	1.62 (1.57‐1.67)	<.001
Parent	1.77 (1.72‐1.83)	1.76 (1.64‐1.88)	2.06 (1.93‐2.21)	1.67 (1.61‐1.74)	<.01
Sibling	2.47 (2.30‐2.67)	3.00 (2.58‐3.49)	2.51 (2.28‐2.76)	2.24 (2.12‐2.36)	.09
Halfsibling	2.59 (2.11‐3.17)	3.12 (2.31‐4.23)	3.17 (2.63‐3.83)	2.07 (1.74‐2.48)	.14
65 and over					
Any relative	1.41 (1.36‐1.45)	1.49 (1.35‐1.64)	1.53 (1.40‐1.68)	1.38 (1.33‐1.43)	.06
Parent	1.43 (1.37‐1.49)	1.50 (1.35‐1.67)	1.55 (1.40‐1.72)	1.36 (1.30‐1.41)	.02
Sibling	1.61 (1.49‐1.74)	1.60 (1.26‐2.04)	1.87 (1.60‐2.19)	1.57 (1.49‐1.65)	.49
Halfsibling	1.77 (1.41‐2.22)	1.51 (1.10‐2.07)	1.78 (1.44‐2.20)	1.65 (1.34‐2.05)	.88
Female					
Any relative	1.55 (1.51‐1.60)	1.59 (1.46‐1.72)	1.83 (1.70‐1.97)	1.50 (1.45‐1.55)	<.0001
Parent	1.61 (1.56‐1.68)	1.61 (1.45‐1.78)	1.99 (1.82‐2.19)	1.55 (1.49‐1.62)	<.0001
Sibling	1.88 (1.73‐2.05)	1.90 (1.31‐2.75)	2.58 (2.09‐3.18)	1.78 (1.62‐1.96)	.01
Halfsibling	2.18 (1.73‐2.74)	2.08 (1.21‐3.58)	2.45 (1.67‐3.60)	2.04 (1.47‐2.85)	.77
Male					
Any relative	1.59 (1.55‐1.63)	1.75 (1.63‐1.88)	1.83 (1.71‐1.95)	1.51 (1.46‐1.56)	<.0001
Parent	1.68 (1.62‐1.73)	1.72 (1.58‐1.87)	1.96 (1.81‐2.11)	1.61 (1.55‐1.67)	<.0001
Sibling	2.05 (1.91‐2.21)	3.07 (2.36‐3.99)	2.25 (1.88‐2.69)	1.96 (1.80‐2.12)	<.01
Halfsibling	2.12 (1.73‐2.59)	2.23 (1.36‐3.63)	2.29 (1.67‐3.13)	1.94 (1.42‐2.65)	.75

Abbreviations: CI, confidence interval; CRC, colorectal cancer; *P*_diff_, *P* value for between country SIR; SIR, standardized incidence ratio.

Across all countries, the SIR for CRC if having any affected relative was similar between females (SIR: 1.55, 95% CI: 1.51‐1.60) and males (SIR: 1.59, 95% CI: 1.55‐1.63). Sex difference in SIR for CRC was most pronounced in the Danish population with SIR for females of 1.59 (95% CI: 1.46‐1.72) compared with SIR for males of 1.75 (95% CI: 1.63‐1.88) whereas the risk was identical for females (SIR: 1.83, 95% CI: 1.70‐1.97) and males (SIR: 1.83, 95% CI: 1.71‐1.95) in Finland (Table 2).

The genetic, childhood environment and shared household environment effects (Table 3) were explored in a model using the observed SIRs for first‐degree relatives by country to calculate RR, presented in Table 2. For individuals below 65 years, the pooled country estimates (RR) were 0.88 (95% CI: 0.53‐1.46) for the first‐degree genetic relatives component, 1.41 (95% CI: 1.26‐1.57) for the childhood environment and 2.08 (95% CI: 1.25‐3.46) for the household environment. For individuals aged 65 or older, the pooled country estimate was 1.04 (95% CI: 0.57‐1.90) for the first‐degree genetic relatives component, 1.14 (95% CI: 0.96‐1.36) for the childhood environment component and 1.41 (95% CI: 0.77‐2.60) for the household environment component (Table 3). This indicates that both childhood environment and household environment are statistically significant contributors to the increased risk of CRC in those with a first‐degree relative, below the age of 65 years with a CRC diagnosis. Notably, across all countries, and in those above and below the age of 65, the genetic contribution to the risk of CRC in first‐degree relatives alone was not found to be statistically significant.

**TABLE 3:** Risk ratio (95% CI) of colorectal cancer in Denmark, Finland and Sweden by shared genetic, childhood and household environment with an affected relative, and age (below 65 and above 65)

Age	Shared with an affected relative^a^	Denmark RR (95% CI)	Finland RR (95% CI)	Sweden RR (95% CI)	*P*_diff_	Pooled RR (95% CI)
Below 65	First degree genetic	0.92 (0.29‐2.98)	0.63 (0.30‐1.30)	1.17 (0.62‐2.21)	.46	0.88^b^ (0.53‐1.46)
	Childhood environment	1.71 (1.30‐2.24)	1.21 (1.02‐1.45)	1.34 (1.21‐1.48)	.13	1.41 (1.26‐1.57)
	Household environment	1.90 (0.59‐6.15)	3.30 (1.58‐6.87)	1.44 (0.76‐2.72)	.25	2.08 (1.25‐3.46)
65 and over	First degree genetic	1.12 (0.28‐4.42)	1.11 (0.44‐2.78)	0.90 (0.42‐1.92)	.93	1.04 (0.57‐1.90)
	Childhood environment	1.07 (0.69‐1.64)	1.21 (0.90‐1.62)	1.16 (1.05‐1.27)	.89	1.14 (0.96‐1.36)
	Household environment	1.34 (0.34‐5.30)	1.40 (0.55‐3.52)	1.51 (0.71‐3.24)	.99	1.41 (0.77‐2.60)

*Note*: N.B. C, G1, G2 and H are derived from the estimated SIR, according to country, age and family relation by inverse‐variance‐weighting (presented in Table 2).

Abbreviations: C, shared childhood component; CI, confidence interval; G1, genetic component first‐degree relative; G2, genetic component second‐degree relatives; H, shared household component; *P*_diff_, *P* value for between country RR; RR, risk ratio.

^a^ Defined by a simplified model: affected parent/child RR modeled as G1*H; affected sibling is modeled as G1*H*C; affected halfsibling is modeled as G2*H*C.

^b^ There is no restriction for this parameter in the model for the genetic component to be <1 although it is logical that it should be ≥1.

The stratified sensitivity analysis showed that the overall household environment contributed to a statistically significant higher risk of CRC than childhood environment independent of age, cancer site or sex, when excluding contribution from the genetic component calculated for Table 3 (Table 4). The contribution of household environment to the RR of CRC, is particularly increased at RR: 1.82, with a narrow 95% CI of 1.76‐1.89, and differs from the pooled result for household environment presented in Table 3 (RR: 2.06, 95% CI: 1.25‐3.46). We also observe a decreasing pooled RR for CRC with increasing age for both shared childhood environment (<55 years: 1.70, 55‐64 years: 1.31, 65‐74 years: 1.15, >75 years: 0.87) and household environment (<55 years: 2.14, 55‐64 years: 1.57, 65‐74 years: 1.52, >75 years: 1.39).

**TABLE 4:** Pooled risk ratio (95% CI) of colorectal cancer by shared childhood and shared household environment for those with an affected relative by sex, site and age (below 65 years and 65 years and over)

Age at CRC diagnosis	Characteristics		Childhood environment: RRSib/RRPar (95% CI)	Household environment: RRPar/G1 (95% CI)
Below 65	All		1.45 (1.27‐1.66)	1.82 (1.76–1.89)
	Sex	Male	1.40 (1.17‐1.68)	1.86 (1.78‐1.94)
		Female	1.49 (1.22‐1.80)	1.78 (1.69‐1.88)
	Site	Colon	1.46 (1.21‐1.76)	1.92 (1.84‐2.01)
		Rectal	1.41 (1.15‐1.72)	1.74 (1.64‐1.84)
	Age	<55	1.70 (1.40‐2.08)	2.14 (2.04‐2.24)
		55–64	1.31 (1.09‐1.58)	1.57 (1.49‐1.66)
65 and over	All		1.13 (0.96‐1.33)	1.47 (1.40‐1.54)
	Sex	Male	1.23 (1.00‐1.50)	1.45 (1.35‐1.55)
		Female	1.00 (0.77‐1.31)	1.49 (1.38‐1.60)
	Site	Colon	1.14 (0.94‐1.38)	1.52 (1.43‐1.61)
		Rectal	0.90 (0.64‐1.27)	1.41 (1.30‐1.54)
	Age	65–74	1.15 (0.94‐1.41)	1.52 (1.42‐1.63)
		≥75	0.87 (0.58‐1.30)	1.39 (1.28‐1.50)

Abbreviations: CI, confidence interval; CRC, colorectal cancer; G1, genetic component first‐degree relative; G2, genetic component second‐degree relatives; RR, risk ratio; RRPar, risk ratio parent; RRSib, risk ratio sibling.

## 4 DISCUSSION

In this large‐scale Nordic cohort study using nationwide population‐based data from cancer and pedigree registries covering over 2.25 million individuals from Denmark, Finland and Sweden, we find an increased relative risk of CRC in those with an affected first‐degree relative compared with national CRC rates regardless of type of relation (parent, sibling, halfsibling) to the CRC index case. We find that the RR is particularly increased in those below the age of 65 years with an affected sibling or halfsibling. In a model including shared environment (shared household and childhood), the overall genetic contribution to the increased RR of CRC identified in this study is not statistically significant. However, both shared household and shared childhood environment remained statistically significant contributors to the risk of CRC in those with an affected first‐degree relative.

We find an increased RR of CRC in relatives of patients with CRC which is in accordance with former, although smaller, studies in the field. However, it is striking that we observe such a large contribution to the observed risk due to shared environment rather than to shared genetics. This has been suggested in a study based on the Swedish Family Cancer Database, which reported that 13% of the familial risk of CRC was attributable to a genetic component, and that environment was the primary causative component in the familial risk of most cases of CRC.^18^ A subsequent study utilizing the same database, found a similar risk of CRC in the halfsiblings of those affected as in the siblings or children of those affected, in keeping with the findings in this study, increased risk of CRC is maintained in the halfsiblings of those affected, compared with national rates. Authors also found that, when assessing second‐degree relatives as a whole, the risk of CRC was negligible, further emphasizing the apparent contribution of shared environment over genetics for the increased risk of CRC we observe.^19^

The statistically significant increased RR of CRC seen in the children of those affected by CRC that we observe in our data from Finland compared with that of Denmark and Sweden is likely to be due to the relatively lower rates of CRC seen in Finland compared with neighboring Nordic countries.^20^ Observed differences are therefore a reflection of the  use of national cancer incidence rates for the calculation of SIRs in familial risk, with differences in familial CRC cohorts and national CRC rates being particularly pronounced in populations with comparatively low cancer incidence such as that seen in Finland.

The majority of CRC incidence is not linked to unique inherited genetic differences. However, it is important to clarify that our findings of a risk ratio of 0.88 for the genetic contribution to CRC risk should not be understood to mean that being genetically related to an index case is protective of CRC. The uncertainty around this risk ratio includes unity (95% CI: 0.53‐1.46). Instead, we interpret these findings as showing no strong evidence for the contribution of genetics to the risk of overall CRC cases observed in those with affected relative, whereas we do find strong evidence for the contribution of shared household and childhood environment to this risk. Findings from our present study indicate that this is the case even for those with affected family members. One of the many risk factors for CRC is obesity and childhood obesity has been identified as a potential contributor to increased incidence rates of colorectal cancer observed in younger age groups.^21^, ^22^ Observed increased CRC risk might be a reflection of shared environmental risk factors for childhood obesity. In addition to childhood obesity, a recent review on early‐onset colorectal cancer identifies early changes to the microbiotia, stress, antibiotic exposure and sedentary lifestyle as potentially independent (albeit closely correlated with obesity) risk factor for early‐onset CRC.^23^ This highlights that diet and lifestyle changes, particularly during childhood, might play a more important role to play in the prevention of hereditary CRC, than genetic screening alone.

We observed a decreased RR for CRC in the shared childhood and household environment with increasing age. It is unclear whether this is product of fewer cases in those over 75 years captured in the follow‐up time in our registries or an underlying biological change. This may however be a reflection of the overall increased risk of CRC in all older age groups reducing the observed contribution of shared household, and particularly shared childhood environment to the RR of CRC in later years of life.

There are changing trends in cancer incidence by age group seen in the US and Europe,^24^, ^25^ with a decreasing incidence of CRC in older age groups and an increase in younger age groups. Evidence suggests that those with a family history of CRC may be particularly susceptible to the wider environmental changes that have contributed to this trend of increased incidence of CRC in younger age groups overall.^26^

Beyond the contribution of known, measurable and modifiable risk factors to CRC, there is a need for increased recognition and characterization of the wider biochemical, physiological and ecological exposures experienced by certain populations. It is widely understood that many environmental factors such as pollutants, toxins, drugs, nutrients and other stressors (the exposome) can be readily identified using modern “omics” technologies. These exposures may be interacting in subtle and complex ways to contribute an increased risk of colorectal cancer, likely through the initiation and mediation of inflammatory processes in the body.^27^ The development of high‐resolution and high‐throughput technologies integrating multiple ‐omics (such as epigenomics, transcriptomics, proteomics, metagenomics and metabolomics) provides an unprecedented opportunity to investigate the impact of the environment on the manifestation of CRC.^28^ A major “bottleneck” in exposomics research is knowing where to look for important environmental risk factors and the present study suggests that analysis of shared household environment in relation to CRC may be informative.

To our knowledge, this is the first study to explore familial aggregates of CRC using national registry data from three Nordics countries with close to complete individual‐level coverage. The study covered CRC incidence data linked with civil registration system data and multigenerational registry data over 34 years of follow‐up.

Although findings for the extent of contribution of childhood and household environment are statistically significant, there are broad confidence intervals around the point estimates, particularly for the contribution of household environment to CRC risk. This highlights that even a comprehensive, population‐based and multi‐country study such as this one may have limited statistical power to investigate these differences. Additionally, although all the registries included have national coverage, relatives diagnosed with CRC prior to the start of registry inception would not be captured and therefore a small proportion of index cases may not have been linked to a family cohort due to this. However, this would be the case for any cohort at inception and due to the extent and completeness of our data this is not likely to bias our observed findings. It is also possible that the between country differences in ratio of relatives to cases had an impact on the statistical differences identified between countries. Another potential limitation is that identification of CRC index cases may have occurred prior to the start of follow‐up of the familial cohorts, resulting in a risk of survival bias (some cases with aggressive disease may have been missed). The average ratio of cases to relatives varies between countries but again, this is unlikely to impact our finding of consistently lower RR contribution of genetics compared with environmental measures for CRC across all countries. Our cohorts were also matched for age, sex and by country of residence for all analysis, making it unlikely that confounding by non‐shared factors had an impact on the findings of this work. The relatively homogenous ethnic makeup of the majority of the inhabitants of the Nordic region, suggests that the findings from this study may be limited in generalizability to other populations. Finally, it is important to highlight that although findings from this work come from real world data, there are important assumptions underlying the model used to calculate RR for the contribution of environment and genetics, specifically the assumed relation between G1 and G2, and that multiplicative structures are good approximations for the interactions between the effects of genetic, shared childhood environment and shared household environment.

In conclusion, although it is difficult to disentangle shared environment from genetics in familial CRC, our findings indicate that shared environment plays a larger role in the development of CRC than genetics.

**Abbreviations**
CI: confidence interval
CRC: colorectal cancer
ICD: International Classification of Diseases
RR: risk ratio
SIR: standardized incidence ratio

## AUTHOR CONTRIBUTIONS

Rahma Elmahdi is responsible for drafting of the manuscript. Elna C. M. Wennerström, Mikael Andersson, Jan Wohlfahrt, Mads Melbye, Eero Pukkala, Maria Hortlund, Kaisa Silander, Kyösti Sutinen, Tine Jess and Joakim Dillner were responsible for study concept and design. Mikael Andersson, Jan Wohlfahrt, Eero Pukkala, Tine Jess and Joakim Dillner were responsible for data acquisition. Mikael Andersson and Jan Wohlfahrt were responsible for data analysis. All authors were responsible for data interpretation. Rahma Elmahdi and Elna C. M. Wennerström were responsible for initial drafting of the manuscript and Rahma Elmahdi, Eero Pukkala, Tine Jess and Joakim Dillner were responsible for critical revision for important intellectual content. The work reported in the article has been performed by the authors, unless clearly specified in the text.

## FUNDING INFORMATION

This study was supported by the NordForsk Foundation. In addition, Rahma Elmahdi and Tine Jess were supported by the Danish National Research Foundation (Grant No. DNRF148) and Elna C. M. Wennerström was supported by an Oak Foundation Fellowship.

## CONFLICT OF INTEREST

The authors declare no conflict of interest.

## Supporting information


**TABLE S1** Observed and expected number of cases, total person years of follow‐up and crude observed incidence rates by age (below 65 years and 65 years and over) and sex (female and male)

## Data Availability

Data used for analysis is available upon application from the Danish, Finnish and Swedish national registry authorities. Further details and other data that support the findings of this study are available from the corresponding author upon request.
