# Supplementary material for: Shared environment and colorectal cancer: A Nordic pedigree registry‐based cohort study
Source: Int J Cancer. 2022 Jun 22;151(8):1261–9. doi: 10.1002/ijc.34148 (PMC9545319; doi:10.1002/ijc.34148)
Supplement: Supplementary file 1 — TABLE S1 Observed and expected number of cases, total person years of follow‐up and crude observed incidence rates by age (below 65 years and 65 years and over) and sex (female and male) [file IJC-151-1261-s001.pdf]

**Supplementary Data**

**Shared Environment and Colorectal Cancer: A Nordic Pedigree Registry-based Cohort Study**

Rahma Elmahdi, PhD; Christina E. M. Wennerström, PhD; Mikael Andersson, MSc; Jan Wohlfahrt, DMSci; Mads Melbye, DMSci; Eero Pukkala, PhD; Maria Hortlund, PhD; Kaisa Silander, PhD; Kyösti Sutinen[9], PhD; Tine Jess, DMSci; Joakim Dillner, DMSci

**Contents** **Page**

**Table S1.** Observed and expected number of cases, total person years of follow-up, and crude observed incidence rates by age (below 65 years and 65 years and over) and sex (female and male) **2**

**Supplementary Data**

| | Relative CRC | Statistic | Denmark | Finland | Sweden |
|---|---|---|---|---|---|
| Below 65 | Parent | Observed | 738 | 851 | 2775 |
| | Parent | Expected | 427.8 | 413.8 | 1658.7 |
| | Parent | Pyrs (100,000) | 16.272 | 17.097 | 46.950 |
| | Parent | Observed rate | 45.4 | 49.8 | 59.1 |
| | Child | Observed | 26 | 22 | 37 |
| | Child | Expected | 7.3 | 9.2 | 20.6 |
| | Child | Pyrs (100,000) | 0.078 | 0.181 | 0.306 |
| | Child | Observed rate | 333 | 122 | 121 |
| | Sibling | Observed | 56 | 143 | 451 |
| | Sibling | Expected | 18.7 | 57.0 | 201.3 |
| | Sibling | Pyrs (100,000) | 0.465 | 1.612 | 3.604 |
| | Sibling | Observed rate | 120 | 88.7 | 125 |
| | Halfsibling | Observed | 14 | 36 | 41 |
| | Halfsibling | Expected | 4.5 | 11.4 | 19.8 |
| | Halfsibling | Pyrs (100,000) | 0.115 | 0.306 | 0.576 |
| | Halfsibling | Observed rate | 122 | 118 | 71.2 |
| 65 and over | Parent | Observed | 176 | 192 | 1613 |
| | Parent | Expected | 130.3 | 120.1 | 1156.9 |
| | Parent | Pyrs (100,000) | 0.467 | 0.730 | 5.389 |
| | Parent | Observed rate | 377 | 263 | 299 |
| | Child | Observed | 178 | 184 | 589 |
| | Child | Expected | 105.6 | 122.3 | 466.0 |
| | Child | Pyrs (100,000) | 0.298 | 0.553 | 1.602 |
| | Child | Observed rate | 598 | 332 | 368 |
| | Sibling | Observed | 22 | 52 | 514 |
| | Sibling | Expected | 13.7 | 27.7 | 327.8 |
| | Sibling | Pyrs (100,000) | 0.046 | 0.163 | 1.476 |
| | Sibling | Observed rate | 479 | 318 | 348 |
| | Halfsibling | Observed | 13 | 28 | 28 |
| | Halfsibling | Expected | 8.6 | 15.7 | 16.9 |
| | Halfsibling | Pyrs (100,000) | 0.026 | 0.083 | 0.077 |
| | Halfsibling | Observed rate | 492 | 336 | 364 |

**Supplementary Data**

| | | | | | |
|---|---|---|---|---|---|
| Female | Parent | Observed | 386 | 432 | 2004 |
| | Parent | Expected | 240.17 | 216.57 | 1290.93 |
| | Parent | Pyrs (100,000) | 7.894 | 8.465 | 25.816 |
| | Parent | Observed rate | 48.9 | 51 | 77.6 |
| | Child | Observed | 113 | 143 | 420 |
| | Child | Expected | 64.94 | 79.02 | 287.13 |
| | Child | Pyrs (100,000) | 0.244 | 0.187 | 1.268 |
| | Child | Observed rate | 462.5 | 291.8 | 331.2 |
| | Sibling | Observed | 28 | 86 | 431 |
| | Sibling | Expected | 14.75 | 33.36 | 241.68 |
| | Sibling | Pyrs (100,000) | 0.247 | 8.465 | 2.577 |
| | Sibling | Observed rate | 113.3 | 10.2 | 167.2 |
| | Halfsibling | Observed | 13 | 26 | 35 |
| | Halfsibling | Expected | 6.26 | 10.59 | 17.12 |
| | Halfsibling | Pyrs (100,000) | 0.069 | 1.268 | 0.355 |
| | Halfsibling | Observed rate | 187.9 | 139.3 | 98.6 |
| Male | Parent | Observed | 554 | 621 | 2505 |
| | Parent | Expected | 322.64 | 321.14 | 1556.77 |
| | Parent | Pyrs (100,000) | 8.882 | 9.42 | 26.765 |
| | Parent | Observed rate | 62.4 | 66.7 | 93.6 |
| | Child | Observed | 102 | 74 | 260 |
| | Child | Expected | 53.06 | 57.18 | 217.07 |
| | Child | Pyrs (100,000) | 0.146 | 0.267 | 0.702 |
| | Child | Observed rate | 698.2 | 277.7 | 370.4 |
| | Sibling | Observed | 56 | 118 | 583 |
| | Sibling | Expected | 18.23 | 52.47 | 297.84 |
| | Sibling | Pyrs (100,000) | 0.268 | 0.941 | 2.565 |
| | Sibling | Observed rate | 208.7 | 125.4 | 227.3 |
| | Halfsibling | Observed | 102 | 39 | 39 |
| | Halfsibling | Expected | 7.19 | 17.04 | 20.13 |
| | Halfsibling | Pyrs (100,000) | 0.0734 | 0.207 | 0.332 |
| | Halfsibling | Observed rate | 218.0 | 188.3 | 117.5 |
